# Loneliness

**Published:** 1890-10-04

**Authors:** 


### Words of Consolation.

### LONELINESS.

"Why should we faint and fear to live alone, since all alone, so Heaven has willed we die?" says the Christian poet, and yet he owns that it is the common wish and earnest desire of man to have the sympathy of some one person. Our souls dwell so alone, so far apart from every other soul that even those we love the best and who love us, do not "know half the reasons why we smile or sigh." There are a few self-contained natures who like it to be so, and with the Spartan boy let the wolf gnaw their vitals without making a sign. They have no sympathy for others, and hate to be pitied; they say pity is near akin to contempt. And few of us would wish our friends to see into our hearts at all times. Do we not often let rude, bad thoughts reign there and thrust from His throne the love which should be lord of all? If those who esteem us could catch a glimpse of what goes on within us, even at our best moments how they would shrink from us as if from a venomous reptile. "So we might friendless live and die unwept." Happily, God keeps a veil drawn down between us and our fellow men. Whatever our actions may show of what goes on behind it, the full measure of our faults and offences are unknown. There is only One Who can love us while reading our hearts and motives. He loves us, notwithstanding our sins and iniquities.

Our friends see us cheerful, courteous, apparently contented, beautiful as whited sepulchres. What is on the other side? Discontent, pride, bitter thoughts against those who oppose us, and jealousy of those who excel us. We give way to restlessness and regret, and have a vehement longing for what we cannot get. We pine, perhaps, for some one to understand us thoroughly and make our lives to be cheerful and contented. We may even pray God to give us a human heart that shall make our happiness complete. Do not pray too fervently even for such a blessing, lest if the wish be granted, we should make to ourselves an earthly idol, and with our hearts beating in perfect harmony, we should be bound fast; chained to earth by our affections we should forget that here we have no abiding city, and should fail to seek one to come.

For our true happiness, we should be content to do without earthly treasures and try to realise we have for a God, a friend who sticketh closer than a brother, and that He is always ready to pour out His sympathy upon us. He will make excuses for our faults and listen to our sorrows if we will pour them into His indulgent ear. Do not hold aloof from Him, He longs to comfort and soothe us; it is our own fault if we are without One who can give us perfect sympathy, who will never leave us alone. He will put His everlasting arm around us and we shall be lulled to rest. If we are hungry He will be near to feed us with the bread of life. Are we thirsty? He will give us to drink of that living water which shall spring up in us into everlasting life. But to gain this friend, this lover, we must believe in Him and turn to Him with all our hearts. Repent and be sorry for our past sins and have a true intention of amendment of life. When He sees that we are tired of all our old idols, that we are earnestly longing to rid ourselves of them and cling only to Him for succour and safety, He will stretch out His protecting arm and gather us into His bosom. He will, for He created our hearts and knows their bitterness; our joys are His. He is no stranger to our wanderings. He loves us though we have darkened the image in which we were made. The Lord who dwells on high knows us, and loves us better than He knows. He will be within us to strengthen us, without us to guard us, over us to shelter us, beneath us to establish us, before us to guide us, after us to forward us, round about us to secure us.

